# Rock-salt and helix structures of silver iodides under ambient conditions

**DOI:** 10.1093/nsr/nwz041

**Published:** 2019-04-02

**Authors:** Hongyang Huang, Jinying Zhang, Yifan Zhang, Chengcheng Fu, Jialiang Huang, Yonghong Cheng, Chunming Niu, Xinluo Zhao, Hisanori Shinohara

**Affiliations:** 1 State Key Laboratory of Electrical Insulation and Power Equipment, Center of Nanomaterials for Renewable Energy (CNRE), School of Electrical Engineering, Xi’an Jiaotong University, Xi’an 710049, China; 2 Department of Physics, Shanghai University, Shanghai 200444, China; 3 Department of Chemistry and Institute for Advanced Research, Nagoya University, Nagoya 464–8602, Japan

**Keywords:** carbon nanotubes, silver iodides, rock salt, helix, ambient conditions

## Abstract

Many different phase structures have been discovered for silver iodides. The β and γ phases were found to be the most common ones at ambient conditions, while the rock-salt phase was found to be stable under pressures between 400 MPa and 11.3 GPa. Recently, the α phase was demonstrated to be stable under ambient conditions when the particle sizes were reduced to below 10 nm. However, no other phase has been reported to be stable for silver iodides under ambient conditions. Rock-salt and helix structures have been found to be stable under ambient conditions in this study. The structures have been characterized by elemental mapping, Raman scattering, and high-resolution transmission electron microscopy. The stabilities of these structures were also confirmed by molecular dynamics and density functional theory.

## INTRODUCTION

Silver iodides are widely employed in cloud seeding [1], contrast agents [2], thermo-electrical batteries [3], and medicines [4]. The structure of silver iodides has been demonstrated to be temperature and size dependent. The β phase (II, wurtzite structure) is the most stable structure at temperatures under 420 K. The β and γ phases (II′) are usually adopted by silver iodides at ambient conditions, while the α phase (I, body-centered cubic structure) is more stable for silver iodides when the temperature is higher than 420 K. A melt lattice for silver ions was formed so that the silver ions could transfer inside the crystal lattice; this was called a superionic conductor. In addition to the α (I), β (II) and γ (II′) phases existing at low pressure, there are the rock-salt phase (III), disordered rock-salt phase (III′), tetragonal phase (IV), and KOH-type phase (V) located at high pressure in the *p*–*T* phase diagram [5,6]. The rock-salt phase is adopted for silver iodides at low temperatures with pressures within 400 MPa–11.3 GPa [7–10]. The tetragonal phase is adopted for silver iodides at lower pressure than the rock-salt phase, 300–400 MPa [9]. The V phase has a structure similar to potassium hydroxide at a much higher pressure—more than 11.3 GPa [10]. The disordered rock-salt phase is adopted at higher temperatures than the rock-salt phase when the pressure is above 600 MPa [5].

It has been demonstrated that the transition temperature for the α phase decreases with decreasing size of silver iodide crystals, which has been demonstrated to decrease to room temperature [11–15]. However, there is no report about other phase structures of silver iodides. The size of silver iodides can be adjusted to be extremely narrow when encapsulated into the narrow cavities of carbon nanotubes. Different allotropes such as ring-shaped phosphorus [16], diamond nanowires [17], metal nanowires [18], molybdenum disulfide nanowires [19], long carbon chains [20–22], and carbon nanotubes with selective chirality [23–25] have been demonstrated to be produced inside carbon nanotubes.

Herein, we report a way to synthesize rock-salt or helix structures of silver iodides tuned by the inner diameters of carbon nanotubes (CNTs). The silver iodides are encapsulated into multi-walled carbon nanotubes (MWCNTs) with inner diameters of 4–8 nm and single-walled carbon nanotubes (SWCNTs) with inner diameters around 1.4 nm. The structures of the encapsulated silver iodides have been demonstrated by Cs-corrected high-resolution transmission electron microscopy (HRTEM), elemental mapping, and Raman scattering. The stability of those structures has also been calculated in dynamics or in energy through molecular dynamics (MD) and density functional theory (DFT) calculations. A stable rock-salt phase structure has been achieved by silver iodides inside MWCNTs with inner diameters of 4–8 nm, while a helix structure has been obtained inside SWCNTs with inner diameters of 1.4 nm, all under ambient conditions.

## RESULTS AND DISCUSSION

The MWCNTs and SWCNTs used were prepared with a chemical vapor deposition (CVD) method [26] and arc discharge method [27], respectively. The silver iodides were sublimed and self-assembled into pre-evacuated nano-cavities of the carbon nanotubes through a vapor-phase transportation method [28] at 773 K for 48 h.

**Figure 1.**
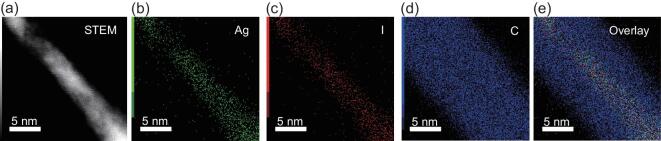
EDS mapping of a carbon nanotube filled with silver iodide. (a) High-angle annular dark-field (HAADF) image. (b–d) Elemental mapping of silver, iodine, and carbon respectively. (e) Overlay mapping of silver, iodine, and carbon.

The successful encapsulation of silver iodides inside carbon nanotubes has been well demonstrated by the elemental mapping of Ag, I, and C (Fig. 1) with a field emission scanning transmission electron microscope (FESTEM). The encapsulated structures have much lighter color compared to the surrounding carbon walls (Fig. 1a) due to the higher atomic numbers of Ag and I than C. The distribution of Ag (Fig. 1b) is comparable to that of I (Fig. 1c), which is fully surrounded by carbon atoms (Fig. 1d and e).

**Figure 2.**
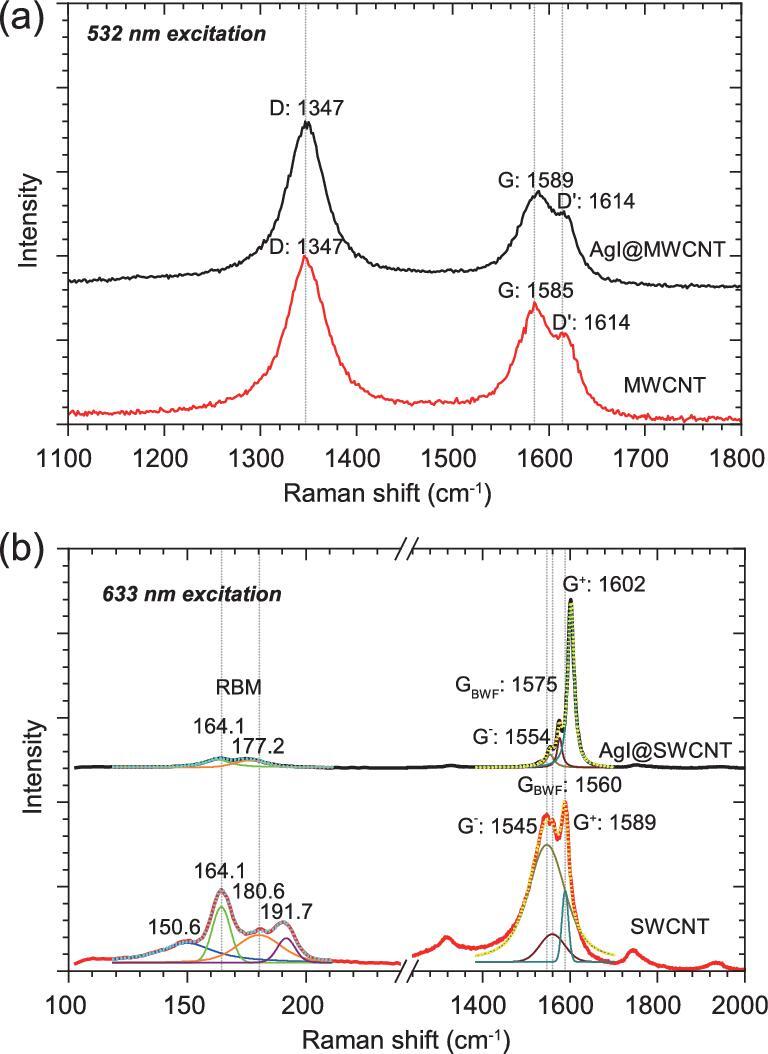
Influence on Raman spectra of carbon nanotubes. (a) The spectra of MWCNTs before (black) and after (red) encapsulation of silver iodide. (b) The spectra of SWCNTs before (black) and after (red) encapsulation of silver iodide, along with their peak splitting analyses (blue, green, orange, purple, and cyan for RBM bands; and dark yellow, claret, dark cyan, and yellow for G bands).

The encapsulation of silver iodides into carbon nanotubes was further confirmed by Raman spectra before and after encapsulation (Fig. 2). The G band of MWCNTs was observed to upshift from 1585 cm^−1^ to 1589 cm^−1^ after encapsulation due to the hole doping of carbon nanotubes (CNTs) [29] as a result of a large difference in the work functions of the compounds and nanotubes (Fig. 2a). The Raman spectra of SWCNTs after encapsulation of silver iodides are easily observed to be different from those of pristine SWCNTs. The Raman spectrum of SWCNTs at the G bands was fitted with the Voigt function and split into three peaks. The G^+^ band, corresponding to the longitudinal optic (LO) mode of semiconducting SWCNTs [30], was observed to upshift from 1589 cm^−1^ to 1602 cm^−1^ (Fig. 2b, dark cyan), while the G_BWF_ band, corresponding to the LO mode of metallic SWCNTs [29, 30], was observed to upshift from 1560 cm^−1^ to 1575 cm^−1^ (Fig. 2b, claret), and the G^−^ band, corresponding to the transversal optic (TO) mode of semiconducting SWCNTs, was observed to upshift from 1545 cm^−1^ to 1554 cm^−1^ (Fig. 2b, dark yellow). The upshifts of the G^+^, G_BWF_, and G^−^ bands are due to the electron transfer from the SWCNTs to the encapsulated silver iodides. The charge transfer has also been calculated to be about 0.44 electrons per simulation unit (612 C atoms, 30 I atoms, and 30 Ag atoms) through a DFT calculation along with Bader charge analysis [31–34]. The corresponding isosurfaces of simulated charge density differences are shown in Supplementary Fig. 1. The LO mode of SWCNTs was observed to be affected by the encapsulation of silver iodides much more than the TO mode, which is consistent with the reported literature [29]. Analogous to the G bands of SWCNTs, the radial breathing mode (RBM) bands were fitted with the Voigt function and split into four peaks. The first and fourth peaks at 150.6 cm^−1^ (Fig. 2b, blue) and 191.7 cm^−1^ (Fig. 2b, purple), corresponding to SWCNTs with chiralities of (20, 1) and (11, 7)/(14, 3), were demonstrated to disappear after the encapsulation of silver iodide (Fig. 2b, purple). The third RBM peak at 180.6 cm^−1^ (Fig. 2b, orange), corresponding to those with a chirality of (13, 6), was observed to have a weaker intensity and to downshift to 177.2 cm^−1^, which might be due to an expansion of the tube diameter. However, the second RBM peak at 164.1 cm^−1^ (Fig. 2b, green), corresponding to those with chiralities of (14, 7) or (18, 1), was observed to have a weaker intensity without any apparent shift. This consequently led to the conclusion that the most suitable tube diameter of SWCNTs for silver iodide encapsulation is between 1.47 nm and 1.34 nm, since the SWCNTs with inner diameters of 1.47 nm did not show any expansions in the Raman spectrum. This means that it may be the most suitable size for the encapsulation procedure.

The Raman spectra of SWCNTs and MWCNTs before and after encapsulation under both 633 nm and 532 nm excitation are presented in Supplementary Fig. 2. An upshift at the G bands was observed after encapsulation. A downshift at the RBM bands was also observed by the SWCNTs after encapsulation. The difference between the G bands of MWCNTs under different excitations originates from the local thermal effect generated by different kinds of excitations.

The encapsulation yields of AgI into MWCNTs and SWCNTs were also evaluated as more than 85% and 90% respectively through conventional transmission electron microscopy (CTEM) in Supplementary Figs 3 and 4. The crystal structures of the silver iodide encapsulated carbon nanotubes were also characterized by X-ray diffraction (XRD) (Supplementary Fig. 5). Two kinds of MWCNTs, one with open caps where the silver iodides were both deposited inside and outside the MWCNTs (Supplementary Fig. 5, black) and the other one without open caps where the silver iodides were basically deposited outside the MWCNTs (Supplementary Fig. 5, red), were used for encapsulation. The XRD patterns are similar to each other, being dominated by the inevitable silver iodides (β/γ) deposited outside the carbon nanotubes. No effective method has been obtained to totally get rid of the outer silver iodides.

However, the phase structures of silver iodides encapsulated inside carbon nanotubes are still unclear. More characterizations are necessary to further explore the phase structures of silver iodides inside carbon nanotubes.

**Figure 3.**
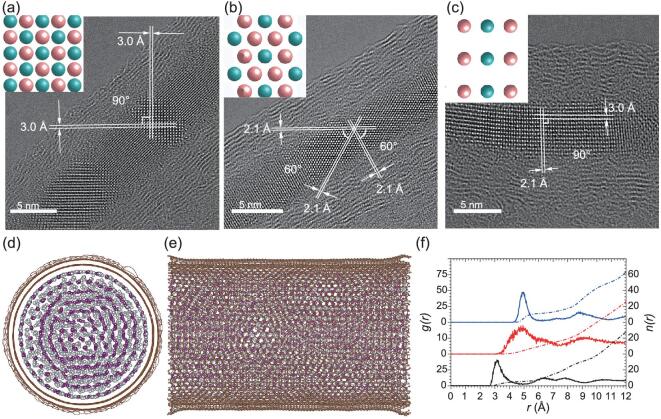
Structure characterization of the silver iodide in MWCNTs. HRTEM images of silver iodide inside MWCNTs viewed along different directions with corresponding structural models. (a–c) HRTEM images with corresponding structural models (inset: red—iodine, green—silver) along <1 0 0>, <1 1 1>, and <1 1 0> respectively. (d, e) The cross section and longitudinal section of the structural model after molecular dynamics simulation. (f) Calculated radial distribution function and average number of neighbors of Ag–I (black line), Ag–Ag (red line) and I–I (blue line) within a sphere of radius *r*.

The silver iodides inside MWCNTs with inner diameters of 4–8 nm were demonstrated by HRTEM and molecular dynamics (MD) simulations to have a rock-salt lattice structure. HRTEM images of silver iodides inside MWCNTs are shown in Fig. 3a–c. Two perpendicular sets of crystal planes with an interplanar crystal spacing of 3.0 Å are observed in Fig. 3a. Both interplanar distances and angles are very consistent with the (0 2 0) and (0 0 2) planes of the rock-salt phase of silver iodides. The HRTEM image is very consistent with the rock-salt structure viewed along <1 0 0> (Fig. 3a, inset). Three sets of crystal planes with an interplanar crystal spacing of 2.1 Å and intersected angles of 60° are observed in Fig. 3b, which is very consistent with the (2 −2 0), (2 0 −2), and (0 2 −2) planes of the rock-salt phase of silver iodides, respectively. The HRTEM image is very consistent with the rock-salt structure of silver iodides viewed along <1 1 1> (Fig. 3b). Another two perpendicular sets of crystal planes with interplanar distances of 3.0 Å and 2.1 Å are observed in Fig. 3c, which is very consistent with the (0 0 2) and (2 −2 0) planes of the rock-salt phase of silver iodides viewed along <1 1 0>. The corresponding structural models are shown in the insets of Fig. 3a–c.

The rock-salt phase of silver iodides inside MWCNTs with inner diameters about 4–8 nm was further confirmed by the simulation results using a MD method [35], where a Parrinello–Rahman–Vashishta (PRV) model [36] was employed to describe the energies between the silver and iodine ions, the Lennard–Jones (LJ) potential [37] was employed to describe the energies between the carbon atoms and silver or iodine ions, and the Tersoff potential [38] was employed to describe the energies between carbon atoms within the carbon nanotubes. A rock-salt phase was obtained for silver iodides encapsulated inside carbon nanotubes with inner diameters of 5.4 nm after annealing and relaxing dynamics, as shown in Fig. 3d and e and Supplementary Fig. 6. A radial distribution function (RDF) was calculated from the structure after MD calculation (Fig. 3f) since the positions of the ions are slightly disordered due to the thermal effect [39]. The same calculation results were obtained when the simulations were also applied to MWCNTs with inner diameters of 4 and 8 nm (Supplementary Fig. 7), confirming that the rock-salt phase was obtained inside MWCNTs with inner diameters between 4 to 8 nm.

The RDF (*g(r)* curves) and the *n(r)* curves of Ag–I, Ag–Ag, and I–I pairs are shown in Fig. 3f. The *n(r)* curve is determined by [40]:

(1) \begin{equation*} {n_{ij}}(r) = 4\pi {\rho _j}\int_{0}^{r}{{{r^2}{g_{ij}}(r)dr}}, \end{equation*}

where *i* and *j* refer to the type ions, *r* refers to the distance of ions *i* from ions *j*, and ρ*_j_* refers to the number of ions *j* in a unit volume. It represents the number of ions *i* in a sphere of radius *r* centered by ions *j*.

The value of *n(r)* at the first valley of *g(r)* refers to the nearest neighbor of *i*–*j* pairs. The average nearest distance of Ag–I pairs was calculated to be 3.15 Å (Fig. 3f, black solid line), slightly bigger than that for the ideal rock-salt phase (3.03 Å) [7,8] due to the size effect of nano-crystals [41], resulting in more surface structures. The nearest distances of Ag–I pairs are 2.83, 2.83, 2.81, and 2.91 Å for the α, β, γ, and tetragonal phases, respectively [10,42,43]. A rock-salt phase was suggested from the calculated nearest distance of Ag–I pairs. On the other hand, the nearest-neighbor number of Ag–I pairs *n*_Ag–I_ is about 5.5 integrated from the *g(r)* curves (Fig. 3f, black dot-dashed line). The nearest-neighbor number of Ag–I pairs is slightly smaller than 6 for the ideal rock-salt phase due to the incomplete edge effects. The nearest-neighbor number of Ag–I pairs is far from 4 for the ideal α, β and γ, and tetragonal phases, further confirming the rock-salt phase structures for silver iodides inside MWCNTs. As for the Ag–Ag and I–I pairs, the nearest distances of the four phases were very close to each other, making it difficult to distinguish them from each other (Fig. 3f, red and blue lines). However, from the *n(r)* curves, the nearest-neighbor number was determined to be 11.5, close to 12 for the ideal rock-salt phase. The nearest-neighbor numbers are 8, 6, and 4 for the α, β, and tetragonal phases, respectively. The nearest-neighbor numbers further confirmed the rock-salt phase for the silver iodides.

**Figure 4.**
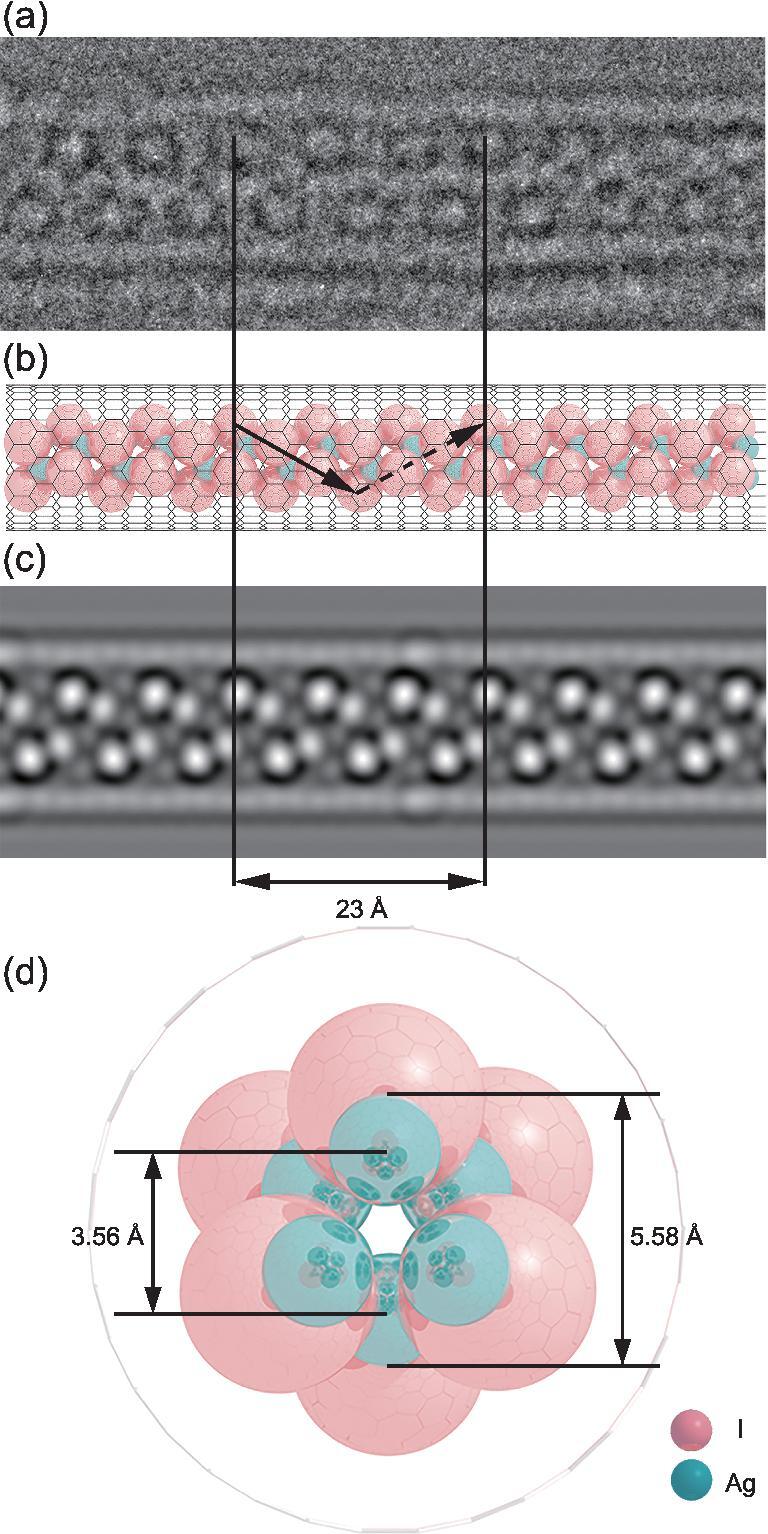
Structure characterization of the silver iodide in SWCNTs. (a) HRTEM image of silver iodides encapsulated inside an SWCNT. (b, d) The longitudinal and cross-sectional structural model of silver iodide inside an SWCNT from the DFT calculation. (c) The corresponding simulated TEM image.

A totally different structure from the α (I), β (II), γ (II′), rock-salt phase (III′), disordered rock-salt phase (III′), tetragonal phase (IV), or KOH-type phase (V) has been observed from the silver iodides encapsulated inside SWCNTs with inner diameters around 1.4 nm. A helix structure was observed from the silver iodides inside SWCNTs by a Cs-corrected HRTEM (Fig. 4a). The helix structure was also obtained by a DFT simulation using the Vienna *ab initio* simulation package (VASP) with exchange correlation function approximated with generalized gradient approximation in Perdew-Burke-Ernzerhof (GGA-PBE) form [44]. The energetically and dynamically stable structure of silver iodides inside an SWCNT with an inner diameter of 1.4 nm is shown in Fig. 4b–d. A triple helix of AgI structure was formed inside SWCNTs with inner diameters around 1.4 nm, where the I ions were located outside of the Ag ions in the radial direction. The diameter of the helix calculated from the I ions is 5.58 Å, while it is 3.56 Å when calculated from the Ag ions (Fig. 4d). The period of the helix along the axial direction is 23 Å. The arrows shown in Fig. 4b are one period of the helix. The simulated TEM image (Fig. 4c) derived from the calculation results shown in Fig. 4b is very consistent with the observed TEM image (Fig. 4a). The observed helix structure of silver iodides is consistent with reported structures inside SWCNTs [45,46]. However, the structure of the silver iodides inside SWCNTs with inner diameters around 1.4 nm [46] was predicted to be twisted hexagonal structure with theoretical confirmation. The twisted hexagonal structure was also calculated by the DFT method with the same parameters to be unstable in this work (Supplementary Fig. 8), further confirming the structure of silver iodide inside SWCNTs as the helix structure.

However, the structure of silver iodides encapsulated inside SWCNTs with inner diameters around 1.4 nm is totally different from those encapsulated inside MWCNTs with inner diameters of 4–8 nm. The formation of different structures of silver iodides inside carbon nanotubes with different diameters are attributed to the size confinement and quasi-pressure effects of the surrounding carbon nanotube walls. Quasi pressures of 1.9–150 GPa have been predicted by the formations of certain crystal structure of KI [47], FePd_3_ [48], Cu [49], Fe [50], and Co [51] inside carbon nanotubes with different diameters. The value depends on the filler structures, temperature and CNT diameters [52]. A quasi pressure of 5 GPa was predicted for nanopores with diameters of 5 nm by grand and semi-grand canonical Monte Carlo simulation [53]. The quasi high pressures provided by carbon nanotubes with diameters of 4–8 nm are between 400 MPa and 11.3 GPa, resulting in the stabilization of rock-salt phases. A quasi pressure around 40 GPa was predicted to be produced inside carbon nanotubes with inner diameters about 1.4 nm [50]. Also, the phase-transition temperatures of high-temperature phases significantly decrease with decreasing size [13]. New phase structures with high temperature and pressures in addition to the exited phase diagram of silver iodides [54] will be formed inside carbon nanotubes with diameters around 1.4 nm, resulting in the formation of helix structures of silver iodides.

The encapsulation method, vaporization in vacuum, was adopted in the formation of different silver iodides. A vapor phase was first produced and then assembled into the carbon nanotubes with different diameters. Different quasi pressures were created inside the carbon nanotubes with different diameters. Different phases in different places of the phase diagram of silver iodides were formed inside carbon nanotubes with different diameters. The nanosize confining effects of carbon nanotubes preserved the as-produced phases at room temperature during cooling to yield the rock-salt and helix structures of silver iodides.

## CONCLUSIONS

Rock-salt and helix structures of silver iodides have been achieved to be stable under ambient conditions by adjusting their diameters using carbon nanotubes with different diameters. A rock-salt phase, which was stable under high pressures of 400 MPa to 11.3 GPa, has been obtained by silver iodides under ambient conditions when the diameters were adjusted to be 4 to 8 nm. A helix structure has been obtained to be stable under ambient conditions when the diameters were decreased to be around 1.4 nm. However, the stable β and γ phases were not observed with narrow diameters up to 10 nm. The silver iodides were self-assembled into rock-salt structures instead of conventional β or γ phases inside MWCNTs with inner diameters of 4–8 nm, while a helix structure instead of any known structures predicted in the *p*–*T* diagram of silver iodides was obtained within SWCNTs with an inner diameter around 1.4 nm. The structure and encapsulation procedure were demonstrated through elemental mapping, Raman scattering, and Cs-corrected HRTEM. The energy or dynamical stability was also verified by MD or DFT calculations.

The properties and applications of the α, β, and γ phases have been well investigated. However, very few studies have been reported for rock-salt or other new phases since the structures are not stable at ambient conditions. The rock-salt and helix structures of silver iodides have been achieved to be stable under ambient conditions in this study, which might lead to detailed research on their properties and applications.

## METHODS

### Synthesis of MWCNTs

The MWCNTs were produced by a chemical vapor deposition (CVD) method with Co/Fe–Al_2_O_3_ as catalyst and ethylene as carbon source. The amorphous carbon and residual metal catalysts were removed from the pristine MWCNTs by heating in air at 773 K for 1 h and then stirring in 5 wt% HF solutions for 24 h. The samples were then filtered with a 0.45 μm membrane film and washed with deionized water. The as-purified MWCNTs were then dried and collected for further experiments.

### Synthesis of SWCNTs

The SWCNTs were produced by a direct current arc discharge method with a graphite rod containing Ni and Y as anode and a pure graphite rod as cathode. A direct current of 90 A was generated between two electrodes under a He protective gas at a pressure of 53.33 kPa. The pristine SWCNTs were collected on the inner walls of the arc discharge instrument.

### Synthesis of AgI encapsulated in MWCNTs

The synthesis of AgI encapsulated in MWCNTs was performed through a vapor method. The purified MWCNTs were heated at 773 K for 30 min under an air atmosphere to open the caps. The open-ended MWCNTs were degassed for one day with interval flame heating and then sealed in the presence of extra silver iodide (Sinopharm, CP) under a vacuum of 10^−5^ Pa in a Pyrex tube. The Pyrex tube was then heated to 773 K for 48 h at a heating rate of 1 K/min and then cooled down in an oven.

### Synthesis of AgI encapsulated in SWCNTs

The synthesis of AgI encapsulated in SWCNTs was performed through a vapor method. The SWCNTs were heated at 693 K for 30 min under an air atmosphere to open the caps. The open-ended SWCNTs were degassed for one day with interval flame heating and then sealed in the presence of extra silver iodide (Sinopharm, CP) under a vacuum of 10^–5^ Pa in a Pyrex tube. The Pyrex tube was then heated to 773 K for 48 h at a heating rate of 1 K/min and then cooled down in an oven.

### Material characterization

Raman spectroscopy was taken in a back-scattering geometry using a single monochromator with a microscope (Reinishaw inVia) equipped with a CCD array detector (1024 × 256 pixels, cooled to 203 K) and an edge filter. The samples were excited by 532 nm and 633 nm argon ion lasers. The spectral resolution and reproducibility was determined to be better than 0.1 cm^−1^. HRTEM images were acquired by FEI TITAN G2 transmission electron microscopy (Cs-TEM; acceleration voltage: 300 kV). CTEM images were acquired by JEM-2100 transmission electron microscopy (TEM; acceleration voltage: 200 kV); elemental mapping of C, I, and Ag was obtained from JEOL JEM-F200 (HR) field emission transmission electron microscopy (FE-TEM; acceleration voltage: 200 kV), equipped with HAADF and EDS modules.

## Supplementary Material

nwz041_Supplemental_File
