# Supplementary material for: Rock-salt and helix structures of silver iodides under ambient conditions
Source: Natl Sci Rev. 2019 Apr 2;6(4):767–74. doi: 10.1093/nsr/nwz041 (PMC8291502; doi:10.1093/nsr/nwz041)
Supplement: nwz041_Supplemental_File [file nwz041_supplemental_file.docx]

**Supporting Information**

**Rock-salt and helix structures of silver iodides under ambient conditions**

Hongyang Huang,^1^ Jinying Zhang,^1*^ Yifan Zhang,^2^ Chengcheng Fu,^1^ Jialiang Huang,^1^ Yonghong Cheng,^1^ Chunming Niu,^1^ Xinluo Zhao,^2^ Hisanori Shinohara^3^

*^1^State Key Laboratory of Electrical Insulation and Power Equipment, Center of Nanomaterials for Renewable Energy (CNRE), School of Electrical Engineering, Xi’an Jiaotong University, Shaanxi 710049, China.*

*^2^Department of Physics, Shanghai University, Shanghai 200444, China.*

*^3^Department of Chemistry and Institute for Advanced Research, Nagoya University, Nagoya 464-8602, Japan.*

*^*^Corresponding author J. Z. E-mail:* [*jinying.zhang@mail.xjtu.edu.cn*](mailto:jinying.zhang@mail.xjtu.edu.cn)

**The MD simulation for AgI encapsulated in MWCNTs**

Three types of potentials, Tersoff, Lennard-Jones (LJ), and Parrinello-Rahman-Vashishta (PRV), were introduced to the calculations. The dynamics of carbon atoms in carbon nanotubes are well described by Tersoff potential. The PRV potential was adopted to describe the interactions of silver iodides since it was usually used for the computing of most of binary ionic crystal structures. It could be written as formula [1]:

$U_{ij}=\frac{q_{i}q_{j}}{r_{ij}}+\frac{H_{ij}}{r_{ij}^{n_{ij}}}-\frac{P_{ij}}{r_{ij}^{4}}-\frac{W_{ij}}{r_{ij}^{6}}$, (1)

Where r*_ij_* is the distance between ions *i* and *j*; *q_i_*, *q_j_* are their effective charges; the parameters *H_ij_*, *n_ij_* describe short-range repulsion, *W_ij_*, is van der Waals attraction, and *P_ij_*, is polarization interactions: $P_{ij}=\frac{1}{2}\left(\alpha_{i}q_{j}^{2}+\alpha_{j}q_{i}^{2}\right)$, α*_i_*, α*_j_* are the polarizabilities of ions *i* and *j*. This approximation was used, successfully enough, in computer modeling of AgI and other silver halide systems, starting from the original work by Parrinello, Rahman, and Vashishta [2].

The potential of Ag and I ions with C atoms could be described with LJ potential, which could be presented as formula [1]:

$U_{ij}=4\varepsilon_{ij}\left[\left(\frac{\sigma_{ij}}{r_{ij}}\right)^{12}-\left(\frac{\sigma_{ij}}{r_{ij}}\right)^{6}\right]$, (2)

The values of parameters are: ε_IC_ = 0.00683 eV, σ_IC_ = 3.736 Å, ε_AgC_ = 0.0335 eV, σ_AgC_ = 2.926 Å [3].

And finally, the dynamics of carbon atoms in carbon nanotubes are described by Tersoff potential, which is widely used to describe the force field between C atoms in carbon nanotubes.

The whole simulation were performed on LAMMPS with a python package for coding the PRV potential. Before the simulation, a 155.56×155.56×98.38 Å^3^ box was built. Then, a double-walled carbon nanotube full filled with silver iodides in gamma phase was put at the center of the box (Fig. S2a). It contained 13610 C atoms, 2685 I ions, and 2685 Ag ions. Enough vacuum space was kept to exclude the disturbance of other MWCNTs, since we employed a whole periodic boundary condition. Besides, the simulation time step was set to 1 fs. After that, the system was relaxed at 300 K for 400 ps firstly, heated to 1000 K in 100 ps, and kept at 1000 K for 400 ps, so that it was fully relaxed. Consequently, it was annealed, as the temperature decreased from 1000 K to 400 K in 100 ps. Next up, it was cooled to 300 K carefully in 400 ps. Finally, it was relaxed at 300 K for 600 ps, and we got a rock-salt phase polycrystal inside the nanotube (Fig. S2b).

With the same method, two additional simulation were performed with MWCNTs of different inner diameters—4 nm and 8 nm, in different but proper sizes of boxes surely, to cover the sizes of MWCNTs used in our experiment. In addition, proper numbers of different types of atoms were introduced, while the average charges kept 0. And finally, the RDF of these 3 types of simulation were drawn in Fig. S3, in which they shared similar features.

**The DFT simulation for AgI encapsulated in SWCNTs**

The initial structure for calculation is shown in Fig. S4a, in which the silver and iodine atoms arranged as Eliseev described [4] in a SWCNT with chirality of (17, 0). The structure of silver iodides inside SWCNTs slowly converged to the final structure, as shown in Fig. 4Sb, with an average binding energy of about -1.78 eV per filled atoms ($\Delta E=\frac{E_{total}-E_{tube}-n_{Ag}\times E_{Ag}-n_{I}\times E_{I}}{n_{Ag}+n_{I}}$). During the simulation, the plane-wave cutoff energy was set to 500 eV. The electronic minimization algorithm was set as RMM-DIIS. The geometric optimization was performed at the Γ point and the projection was performed in the real space to accelerate the calculation since the cell in real space is large enough, which contains 672 atoms. The energy convergence tolerance was set to be 1×10^-‍8^ eV, and the force convergence tolerance was set to be 0.02 eV/Å. And a Gaussian smearing method was adopted with the σ = 0.1 along this calculation.


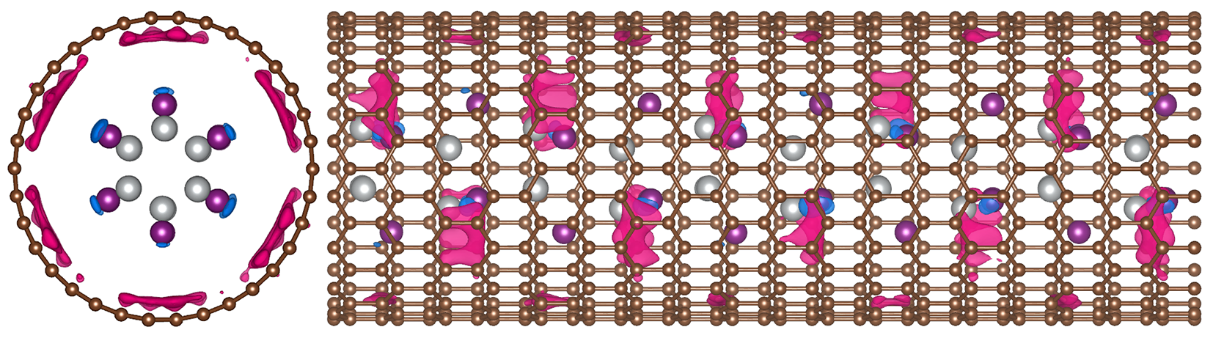


**Fig. S1.** Isosurfaces of charge density differences for AgI encapsulated inside SWCNT. The red and blue colors correspond to the electron depletion and accumulation regions respectively, for a value for the isosurfaces of 0.0006 eÅ^-3^.


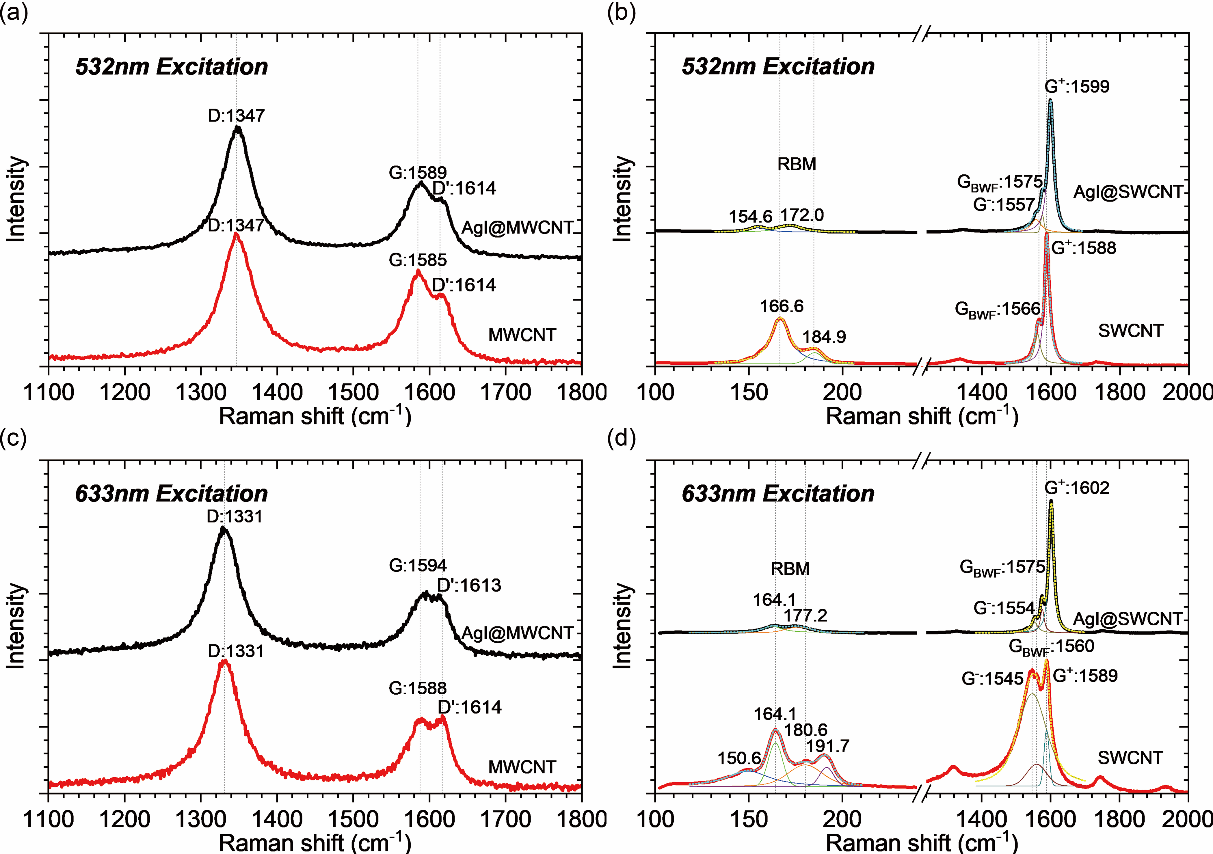


**Fig. S2.** Raman spectra of SWCNT and MWCNT with and without encapsulation under different excitations. (a) The Raman spectra of MWCNTs with and without encapsulation under the excitation of 532 nm laser, in which the G band slightly upshifted after encapsulation. (b) That of SWCNTs under the excitation of 532 nm laser, in which the RBM and G bands were split with Voigt function, and all the G bands upshifted, while all the RBM bands downshifted. (c) That of MWCNTs under the excitation of 633 nm laser, in which the G band also upshifted slightly. (d) That of SWCNTs under the excitation of 633 nm laser, the G bands all upshifted, while the RBM bands mostly downshifted.


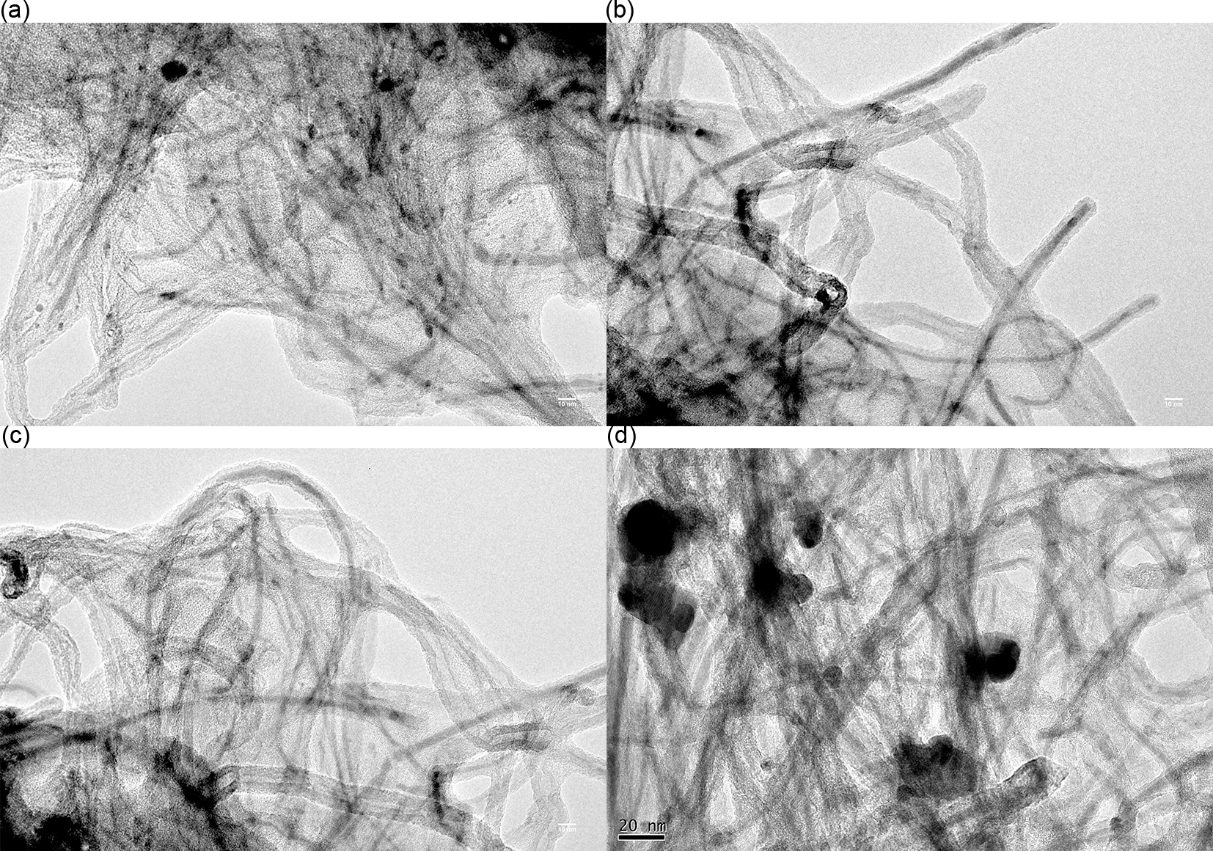


**Fig. S3.** The encapsulation of AgI into MWCNTs. The yield is more than 85%.


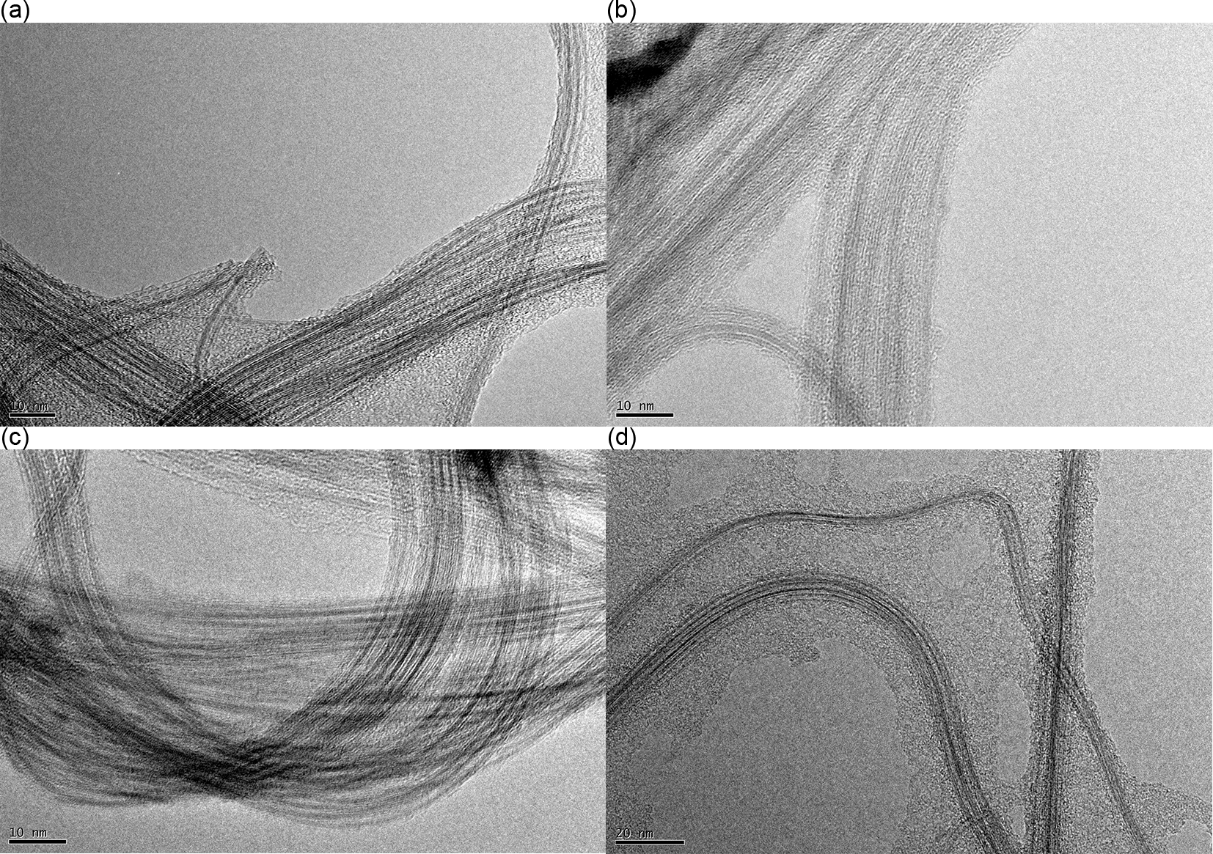


**Fig. S4.** The encapsulation of AgI into the SWCNTs. The yield is more than 90%.


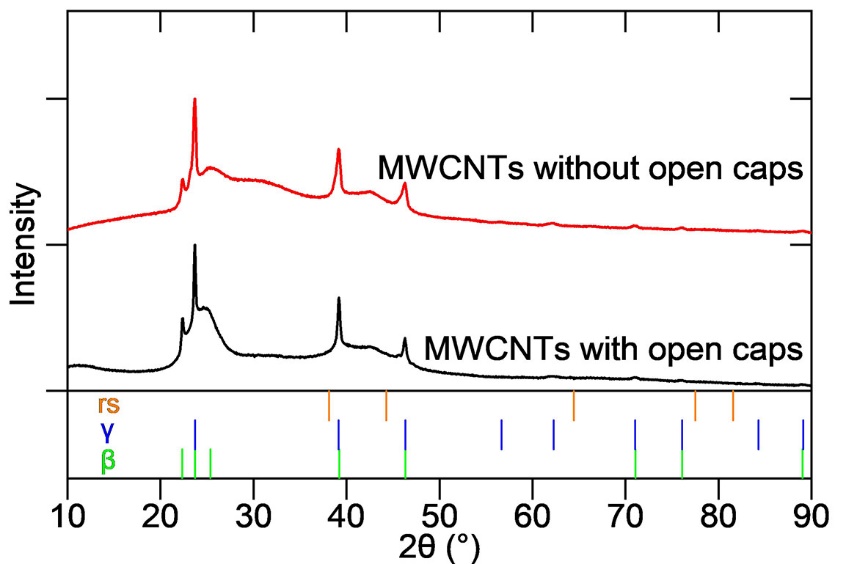


**Fig. S5.** The XRD patterns of AgI encapsulated in two kinds of MWCNTs, one with open caps (black) and the other without open caps (red).


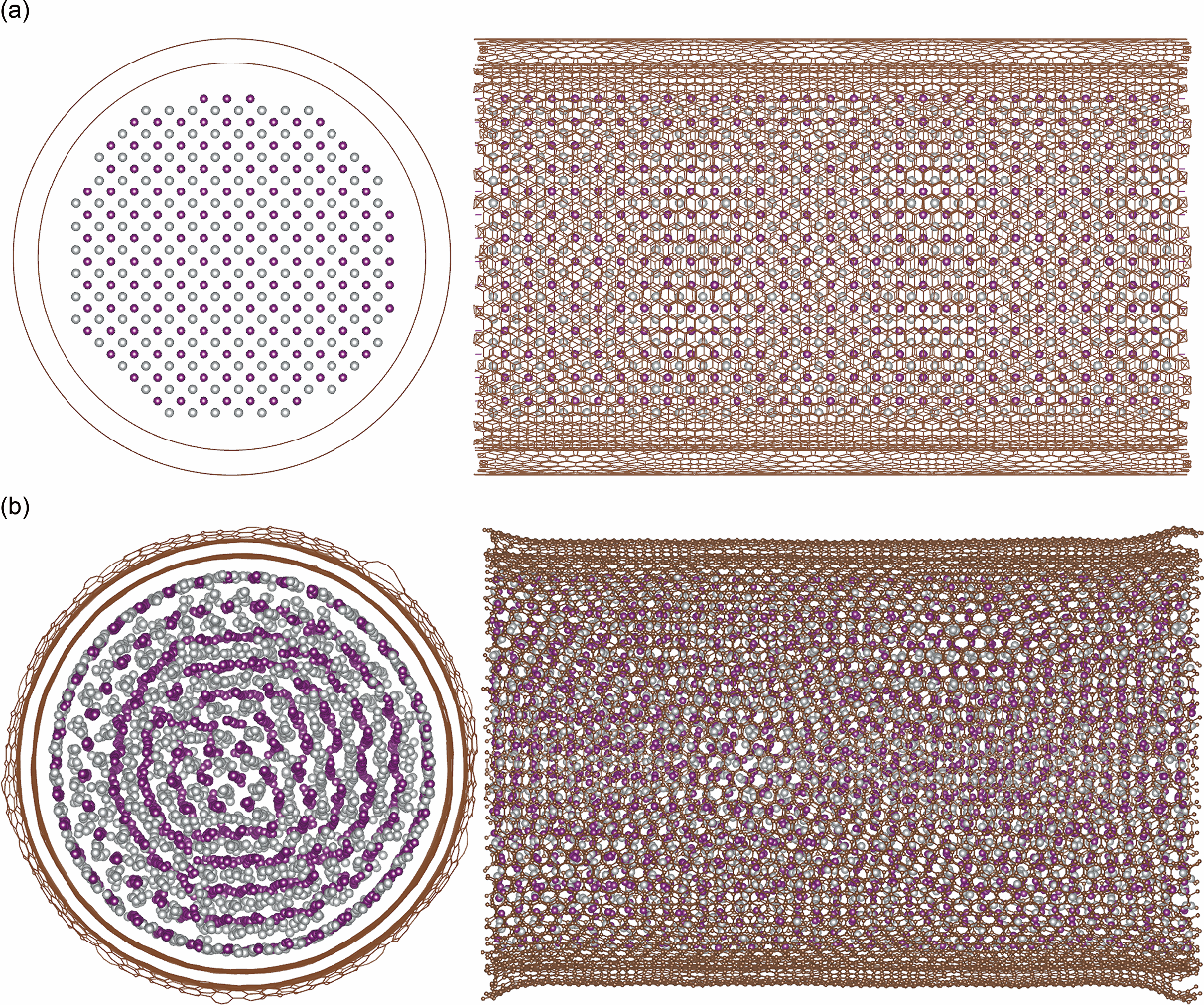


**Fig. S6.** The simulated structure of silver iodides inside MWCNTs through MD methods. (a) The initial structure for MD simulation. (b) The final annealed and relaxed structure for MD simulation.


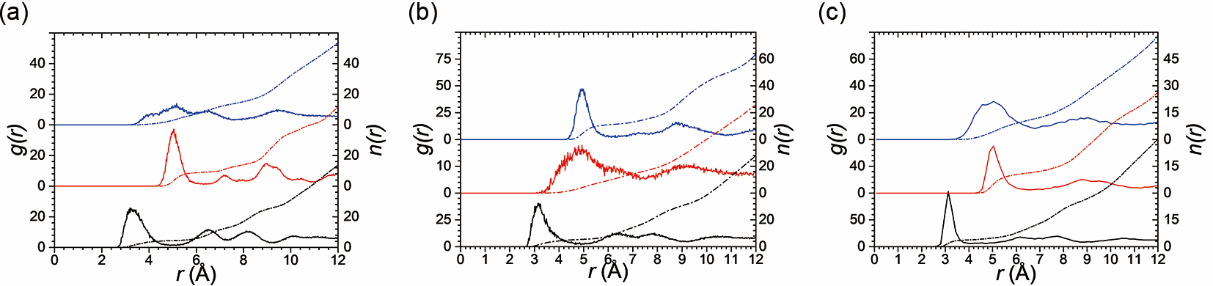


**Fig. S7.** Simulated RDF of silver iodides in different size of MWCNTs. (a) MWCNTs with an inner diameter of 4 nm. (b) MWCNTs with an inner diameter of 5 nm. (c) MWCNTs with an inner diameter of 8 nm.


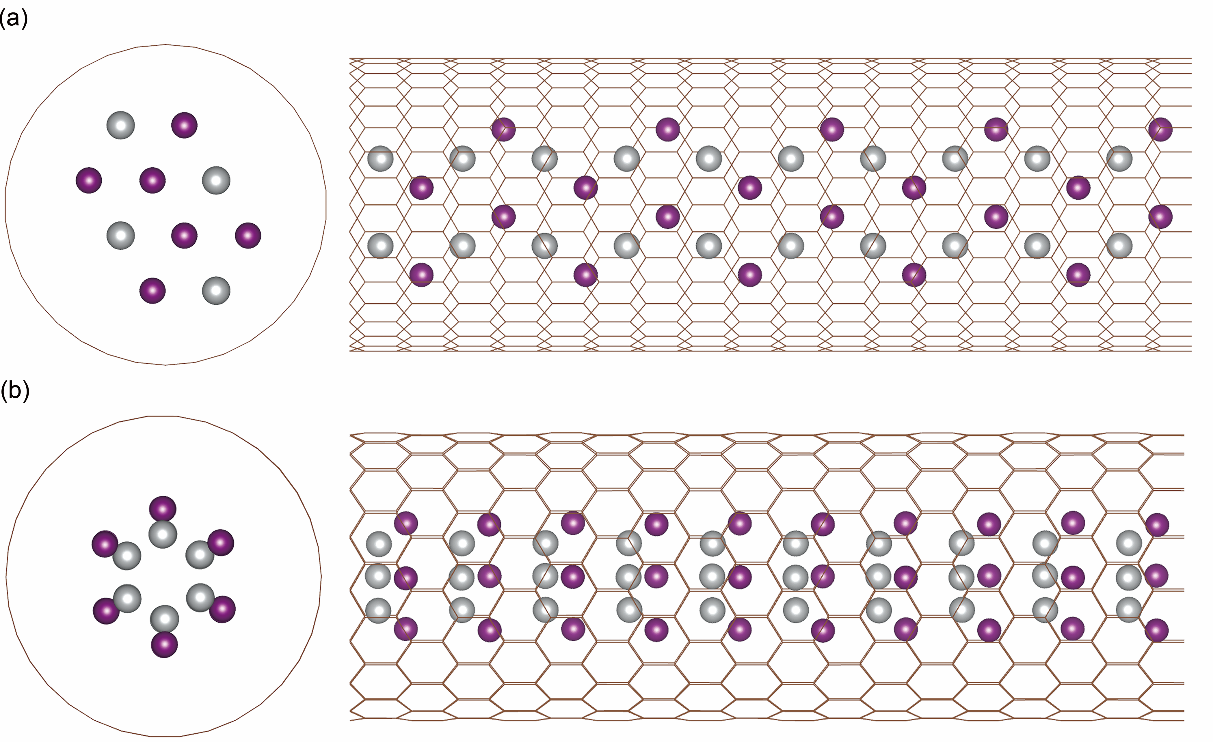


**Fig. S8.** The optimization of silver iodide inside SWCNTs through DFT computing. (a) The initial structure of silver iodides in SWCNTs—a twisted hexagonal structure. (b) The optimized final stable structure both in energy and in forces—a triple helix for silver and iodine ions respectively.

**Reference**

1. Gotlib, IY, Ivanov-Schitz, AK, Murin, IV*, et al.* Computer simulation of ionic transport in silver iodide within carbon nanotubes. *Solid State Ionics*. 2011; **188**(1): 6-14.

2. Parrinello, M, Rahman, A, Vashishta, P. Structural Transitions in Superionic Conductors. *Physical Review Letters*. 1983; **50**(14): 1073-6.

3. Baldoni, M, Leoni, S, Sgamellotti, A*, et al.* Formation, Structure, and Polymorphism of Novel Lowest-Dimensional AgI Nanoaggregates by Encapsulation in Carbon Nanotubes. *Small*. 2007; **3**(10): 1730-4.

4. Eliseev, AA, Yashina, LV, Brzhezinskaya, MM*, et al.* Structure and electronic properties of AgX (X=Cl, Br, I)-intercalated single-walled carbon nanotubes. *Carbon*. 2010; **48**(10): 2708-21.
